# Environmental Parameters as Indicators of Bear Welfare

**DOI:** 10.1002/zoo.70010

**Published:** 2025-07-14

**Authors:** Anna Fourage, Chris R. Shepherd, K. A. I. Nekaris, Vincent Nijman

**Affiliations:** ^1^ Department of Social Sciences Oxford Brookes University Oxford UK; ^2^ Center for Biological Biodiversity Big Lake Ranch British Colombia Canada; ^3^ IUCN SSC Bear Specialist Group Gland Switzerland; ^4^ Anglia Ruskin University School of Life Sciences Cambridge UK

**Keywords:** animal welfare, captive wildlife, Southeast Asia, welfare assessment, zoological associations

## Abstract

Bears are commonly displayed in zoos worldwide. Historically, bears have been housed in poor conditions and even now can be found in inhumane bear pits despite an increased knowledge of animal welfare and husbandry practices. In many developing countries, regular and comprehensive welfare assessments are rarely conducted, especially if not required or enforced by regulatory bodies. A majority of zoos in these countries are also not members of zoo associations. To this end, we focused on evaluating bear exhibits in Thailand, a country with numerous zoos, weak regulations and enforcement pertaining to captive wildlife. We developed a simple assessment of front‐of‐house conditions using environmental parameters to evaluate whether zoos are providing appropriate enclosures as an indicator of welfare potential. We found 77 bears of four species on display in 37 exhibits in 17 zoos (6 accredited, 6 government and 5 private). Our results suggest that more than half of bears displayed in Thai zoos experience poor welfare in exhibits that do not meet basic standards. Overall, 49% of the 37 exhibits were below minimum size, with 54% having no vegetation and 62% having no enrichment. Accredited zoos had significantly better exhibits than government and private zoos. Our research highlights the need for the implementation of zoo standards, in addition to strengthened regulatory measures and enforcement regulating the care of captive wildlife in Thailand.

## Graphical abstract

More than half of the bear exhibits in Thai zoos do not provide the environmental resources needed for good welfare potential. Accredited zoos provided better exhibits than government and private zoos.

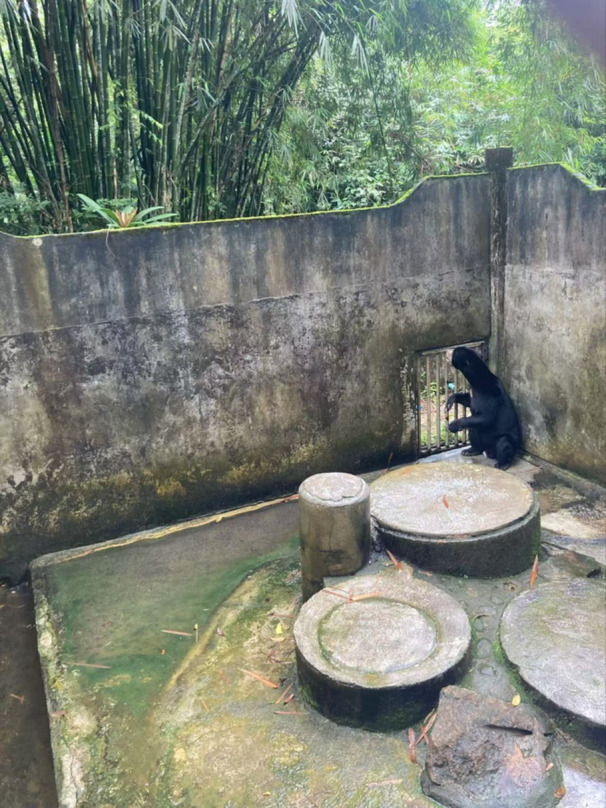

## 1 Introduction

Zoos in the 21st century are increasingly expected to provide high standards of animal welfare as knowledge of animal welfare and husbandry has increased in addition to the societal expectations on the treatment of animals (Fa et al. 2013; Gray 2017; Hampton and Teh‐White 2019; Veasey 2022). Some zoos have joined zoological associations to actively contribute to the zoo community and benefit from the social license that accreditation status may confer. In some countries, zoo associations may require higher animal welfare standards than legislatively required, which is especially true in countries that may not have robust legislation protecting captive wildlife (Irwin 2003; Carr and Broom 2016; Gray 2017). As it stands, a majority of zoos around the world remain non‐accredited (Barber et al. 2010; Ward et al. 2020) and can be considered ‘dysfunctional’ in the sense that they do not operate according to the established principles of a modern zoo (Walker 2011), for example, making a contribution to conservation, education, research and the provision of good animal welfare (see Rose and Riley 2022). Walker (2011) claims that these zoos often do more harm than good in relation to achieving modern zoo objectives. Consequently, the level of care afforded to captive wildlife in many unaccredited zoos may be inadequate.

One crucial aspect of managing captive wildlife is the frequent assessment of animal welfare (Jones et al. 2022). There are several theoretical models of animal welfare, including the Five Freedoms (Brambell 1965), the Five Domains model (Mellor et al. 2020), and the Animal Welfare Assessment Grid (Honess and Wolfensohn 2010). Approaches to assessing welfare vary and should ideally encompass multiple methods (Broom 1991; Maher et al. 2021). Animal‐based measures include physiological indicators (e.g., heart rate, faecal glucocorticoids) and behavioural indicators (Wolfensohn et al. 2018). Resource‐based measures consider indicators such as space, furnishings and the provision of enrichment (Whay 2007; Whitham and Wielebnowski 2013). Welfare assessments typically place more emphasis on animal‐based measures, whilst less emphasis is placed on the affective states of animals, that is, how the animal feels, resulting from their physical and social environments (Veasey 2017). A physically healthy animal does not necessarily mean it is also in a positive mental state, especially if housed in species‐inappropriate and unstimulating environments.

Utilising several welfare measures may be the norm for zoos that adhere to zoo association and regulatory standards, yet the reality is that for most zoos globally, multiple measures are not commonly used. In fact, animal welfare definitions are not widely agreed upon even amongst key welfare stakeholders, including the zoo community (Veasey 2022), and vary across cultures (Appleby and Hughes 1997; Fraser 2008). The resources needed to measure and analyse animal‐based measures in many zoos may be limited. Issues are compounded as the latest evidence‐based insights in the scientific literature are primarily available in English (Ward et al. 2020) or are inaccessible if not published open access (Arunachalam 2004). Additionally, not all zoos (especially non‐accredited zoos) are willing to cooperate with external welfare assessors, especially if it is not legislatively mandated.

There are bear welfare tools and assessments that have previously been developed, including a questionnaire for zoos housing bears in Poland (Maślak et al. 2016) and a keeper‐ratings assessment tool (Maher et al. 2021). These are all typically completed by having access to all areas of the zoo and all parts of the exhibit and records of the animals. It is necessary to develop an assessment that can be conducted without this, similar to methods previously used to measure welfare in the wildlife trade (Nekaris et al. 2016; Bergin and Nijman 2019), zoos and wildlife tourist attractions that are based on resource provisions (Corrigan et al. 2010; Schmidt‐Burbach et al. 2015; Fourage et al. 2022; Fourage, Nekaris, et al. 2024). Such assessments can be applied in real‐world situations that recognise limitations in terms of time, money and access to animals (see Browning 2022). Importantly, these assessments should be conducted by experienced assessors. The limitations of assessing welfare using resource‐based measures alone are well established (Whitham and Wielebnowski 2013; Cole and Fraser 2018). However, evaluating the environmental parameters of an animal's on‐show exhibit can be an indicator of welfare potential, defined as ‘the potential that animals will experience good care based on the care that they are provided with’ (Barber 2009, 519) may be preferable to no assessment at all.

A fundamental factor influencing a captive animal's quality of life is the quality of its housing and exhibit design (Shepherdson et al. 1998; Hosey et al. 2013; Coe and Dykstra 2010). Exhibit designers must comprehensively understand the species' biology, ecology and behaviour to determine what the animal needs to fulfil species‐specific behaviour. Most primates, for example, need enclosures that utilise horizontal and vertical space to provide for their arboreal nature, facilitating locomotion through the exhibit without the animal's being on the ground (Farmer et al. 2023). Thus, all zoo exhibits should be designed to reflect the species' natural history and provide animals with choice and control within their environment (Morgan and Tromborg 2007; Podturkin 2022).

One example where knowledge of exhibit design and animal management has improved over the last few decades is among bears (Ursidae). Bears have a known ability to escape exhibits due in part to their intelligence and destructive nature (Law and Reid 2010; Tabellario et al. 2020), which contributed towards their being kept in pit‐style exhibits that were often barren spaces unable to meet their welfare needs (Law et al. 1992; Tan et al. 2013; Maślak et al. 2016). Responsible zoos have generally moved away from displaying bears in such a way as knowledge of animal welfare and appropriate species husbandry increased, coupled with legislative reforms and zoo association standards (Laidlaw et al. 2010). For instance, as bears are thought to display the highest incidences of stereotypies of carnivore species (Mason 1991; Clubb and Mason 2003; Mason and Latham 2004), well‐managed enrichment programmes can better provide for their complex needs (Bauer et al. 2013; Soriano et al. 2016). Furthermore, exhibits in welfare‐orientated zoos are now designed so that the bear can be elevated to visitor level or, at a minimum, to the same level to help prevent stress (Coe 1985).

Six of the eight bear species are listed as globally threatened on the International Union for Conservation of Nature (IUCN) Red List. We focus on Thailand, which has two native bear species, the Asiatic black bear (*Ursus thibetanus*) and the Malayan sun bear (*Helarctos malayanus*). Thailand has a large number of zoos of varying types and quality (Walker 2022; Fourage, Erzinclioglu, et al. 2024) that can be categorised as follows: (1) accredited zoos under the regional Southeast Asian Zoos Association (SEAZA). All accredited zoos in Thailand except one are run by the Zoological Parks Organisation Thailand (ZPOT); the other zoo is managed by a development agency under contract by local government; (2) government zoos comprised of both local zoos and small open zoos affiliated with the Wildlife Rescue and Breeding Centres under management of the Department of National Parks, Wildlife and Plant Conservation (hereafter referred to as the Department of National Parks); (3) privately owned zoos that are open to the public that predominantly operate for‐profit frequently offering exploitative human–animal interactions (Fourage, Erzinclioglu, et al. 2024). Although Thailand has some legislation pertaining to captive wildlife, it is ambiguous. The Wild Animal Preservation and Protection Act BE 2362 (2019) (WARPA) does not detail specific requirements for wild animals in captive conditions, nor does The Animal Welfare Act BE 2557 (2014) stipulate the meaning of welfare or clearly define terms (Dorloh 2017). Therefore, comprehensive standards for housing captive bears are not mandated.

We aimed to (1) evaluate bear exhibits using a practical scoring system to identify welfare potential for captive bears and (2) evaluate whether there is a correlation between exhibit quality and the type of zoo. A previous study on hornbill (Bucerotidae) showed that welfare was significantly higher in accredited zoos than in other zoo types (Fourage et al. 2022). Hence, we expected that the bear exhibits in accredited zoos would score higher than those of government and private zoos, as accredited zoos are obligated to meet zoo association standards. To the best of our knowledge, this study provides the first report using environmental parameters to assess the welfare potential of bears in Thai zoos. Such data are vital to identify areas for improvement, highlighting the need for zoo standards and the importance of zoo accreditation.

## 2 Methods

### 2.1 Zoo Surveys

Between July 2020 and October 2023, we surveyed 55 zoos throughout Thailand to identify whether bears were on display. We only visited zoos that are open to the public and assessed on‐show exhibits without evaluating back‐of‐house conditions. We subsequently identified 17 zoos that displayed bears and conducted exhibit assessments. The COVID‐19 pandemic resulted in the closure of several private zoos and the temporary closure of several of the open zoos attached to the Wildlife Rescue and Breeding Centres that are part of the Department of National Parks. There are reportedly 26 Department of National Parks facilities (Anon 2022), most of which are closed to the public. The study was purely observational in nature and was added to Oxford Brookes University's Register of Activities Involving Animals, and was reviewed and approved annually over the period 2020–2023. The research was conducted in compliance with Monitor Conservation Research Society's Code of Ethics.

### 2.2 Development of the Exhibit Assessment

We initially reviewed the bear biology and behaviour literature and selected environmental parameters to form the assessment (Supporting Material S1). We also consulted the American Zoological Association (AZA) *Sun Bear and Sloth Bear Care Manual* (AZA Bear Taxon Advisory Group 2019), *The Care and Husbandry Manual for Asiatic Black Bears and Malayan Sun Bears* from Animals Asia (Animals Asia 2017) and the *Standards for Bear Sanctuaries* by the Global Federation for Animal Sanctuaries (Global Federation of Animal Sanctuaries (GFAS) 2013) to assist in the development of the scoring framework. We assigned a score of 0, 1 and 2 to each parameter, with a maximum overall score of 28 available per exhibit (Table 1).

**Table 1:** Assessment scoring criteria used for bear exhibits in 17 zoos in Thailand from July 2020 to October 2023.

	Score		
Measure	0	1	2
Exhibit size	Exhibit is too small (< 279 m^2^)^a^	Exhibit is medium‐sized (279–465 m^2^)^a^	Exhibit is large (> 465 m^2^)^a^
Exhibit topography	Exhibit is completely flat, and vertical space is not utilised.	Exhibit has some variation in topography—e.g., not totally flat through natural variations or by exhibit design (furniture)	Exhibit has well‐designed variations in topography, e.g., hills, rocky outcrops, and tall climbing structures that provide multi‐levelled pathways
Shelter and shade	Animal faces direct exposure to the weather without shelter or shade, or the exhibit is wholly covered	Animal has some shelter or shade, but this is limited to one or two areas or insufficient to allow all animals within the exhibit to seek shade, or the exhibit is 100% covered but open on all four sides	Exhibit is open‐air air, with all animals having the choice to access to shelter or shade in different parts of the exhibit
Permanent furniture	Exhibit is largely barren	A few items of species‐specific furniture, but not a wide range and not enough for all animals	A good variety of species‐specific furniture is enough for all animals housed within
Substrate	Only unsuitable substrates, e.g., concrete, tile	A mix of unsuitable and suitable substrates substrate, e.g., concrete and grass	A variety of suitable substrates, e.g., grass, soil, sand, mulch
Vegetation	No vegetation	Some vegetation in the exhibit	Extensive vegetation throughout the exhibit
Cleanliness	Unhygienic exhibit, e.g., a significant quantity of discarded food, faeces, and litter. Food inappropriately placed on the floor	Moderately clean exhibit, e.g., no litter, but evidence of a couple of days' worth of discarded food and faeces build‐up	Clean exhibit, e.g., no litter, any food, or faeces is from the same day
Ventilation	Exhibit has little to no ventilation, e.g., indoors/fully enclosed space with no evidence of ventilation	Exhibit sides have some tall walls with average ventilation	Exhibit is outdoors and open air with natural ventilation
Light	Animal housed indoors with artificial lighting and no access to or very limited natural light or in an outdoor enclosure with insufficient natural light	Access to some natural light, but this is limited in intensity and not in multiple parts of the exhibit	Access to natural light in multiple areas of the exhibit
Environmental noise	Immediate vicinity to frequent loud noise, e.g., electronic noise from entertainment shows, public address systems	Some electronic noise, e.g., background music, but not too loud	Electronic noise cannot be heard over natural sounds
Exhibit barrier	The barrier is not secure. Barrier material may cause injury to the animal. Visitor/animal interactions are possible due to the absence of a guardrail	Barrier prevents escape, but guardrails are not used to prevent visitor/animal interactions	The barrier is secure, and guardrails are used to prevent visitor/animal interactions
Enrichment	No items that can be manipulated for play and promotion of natural behaviour	One to two items that can promote play and natural behaviours	A variety of three or more items that promote play and natural behaviours
Visual barriers	Animal can be viewed on more than two sides of the enclosure and/or is fully exposed to visitors in all areas of its exhibit and/or animals in neighbouring exhibits. Animal is unable to retreat from conspecifics	Animals can hide from the public but cannot hide from conspecifics or congenerics in neighbouring exhibits	All animals have multiple options of places to retreat fully from conspecifics and fully hide from visitors using vegetation, shrubs, rocky structures
Appropriate social grouping	Animal is housed alone or is housed with an excessive number of animals for the size and resources available within the exhibit	The animal is not housed alone, but the available resources are not ideal for the number of bears	The number of bears in the exhibit is appropriate for the size and available resources

a Size is from the AZA Bear Taxon Advisory Group's (2019) *Sun and Sloth Bear Care Manual* as the minimum sizes given here are the lowest out of the consulted husbandry manuals.

### 2.3 Conducting Assessments

Each exhibit took no more than 5 min to assess. One assessor took videos on an iPhone, and they gave an oral description of each exhibit to assist in completing the scoring framework post‐visit. The number of bears was counted from what could be observed within the exhibit without looking at back‐of‐house areas. Scoring was completed on an Excel spreadsheet. To enhance data reliability, a co‐author conducted onsite visits to 4 out of the 17 zoos included in the study. They also watched a subset of videos of zoos that they did not attend and independently gave a quantitative score for the assessments with an average of 96% agreement.

### 2.4 Analysis

As none of the data were normally distributed for the statistical analysis, we used non‐parametric statistics to assess differences in welfare scores between the three zoo types. We first used a Kruskal–Wallis test, followed by Mann–Whitney *U* tests, to test for differences across zoo types and all the assessment measures. We excluded the water variable and the variable for whether bears had access to a back‐of‐house area from quantitative analysis due to some missing values, as some of the exhibits were so large that we could not view the entire exhibit. Instead, we report these variables in the qualitative description of each exhibit, including whether or not they were observed. We report means ± 1 standard error and took *p* < 0.05 as the significance level in a two‐tailed test. We used IBM SPSS (version 28) for data analysis.

## 3 Results

We assessed 37 bear exhibits, comprising 15 in 6 accredited zoos, 13 in 6 government zoos and 9 in 5 private zoos. We observed 77 bears of 4 species, comprising 74 adults and 3 juveniles, on display (Table 2). The Asiatic black bear and Malayan sun bear accounted for 90% of bears observed. Only 10% of bears were non‐native, including the American black bear (*Ursus americanus*) and the sloth bear (*Melursus ursinus*). We observed the enclosure of a single giant panda (*Ailuropoda melanoleuca* [*A. melanoleuca*]) on 21 April 2023 in an accredited zoo, but the animal itself had died just 2 days before, so we excluded this enclosure from the analysis.

**Table 2:** Table showing the number of bears and their species observed by zoo type (number of zoos in parentheses).

Species	Accredited	Government	Private	Total
Asiatic black bear (*Ursus thibetanus*)	13 (6)	13 (6)	21 (5)	47
Malayan sun bear (*Helarctos malayanus*)	17 (6)	5 (4)	0 (0)	22
American black bear (*Ursus americanus*)	0 (0)	0 (0)	4 (1)	4
Sloth bear (*Melursis ursinus*)	2 (1)	0 (0)	2 (1)	4
Total	32	18	27	77

### 3.1 Exhibit Assessment

The average score per exhibit was 16.00 (SE 1.28) out of a possible score of 28, with a wide range (5–27) of scores (Table 3). Accredited zoos scored the highest in our assessment, with a mean score of 24.00 (SE 0.74), followed by private zoos with a mean score of 11.89 (SE 2.07) and government zoos with a mean score of 9.62 (SE 0.87). There was a significant difference between zoo types (Kruskal–Wallis *H* = 24.941, df = 2, *p* < 0.001). Accredited zoos were significantly better than government zoos (Mann–Whitney *U*, *Z* = −4.517, *p* < 0.001) and private zoos (*Z* = −3.742, *p* < 0.001). There was no significant difference in the scores between government and private zoos (*Z* = −0.545 *p* = 0.586) nor a difference between exhibit scores and species. The highest‐scoring assessment measure was access to natural light, whereas the provision of enrichment scored the lowest (Table 3).

**Table 3:** Assessment measures in rank order of the highest score by mean overall score per measure for 37 exhibits and mean score by zoo type and SEM in parentheses.

Assessment criteria	Mean score per measure across 35 exhibits	Accredited (A) (15 exhibits)	Government (G) (13 exhibits)	Private (P) (9 exhibits)	Sig difference
Light	1.73 (0.10)	2.00 (0.00)	1.69 (0.18)	1.33 (0.29)	a
Barrier	1.70 (0.11)	1.87 (0.13)	1.77 (0.12)	1.33 (0.33)	a
Ventilation	1.65 (0.80)	2.00 (0.00)	1.46 (0.14)	1.67 (0.17)	A > G, P
Cleanliness	1.46 (0.13)	2.00 (0.00)	0.85 (0.25)	1.44 (0.24)	A > G, P
Shade	1.32 (0.10)	1.87 (0.09)	0.77 (0.12)	1.22 (0.15)	A > G, P
Noise	1.24 (0.15)	1.40 (0.21)	1.85 (0.10)	0.11 (0.11)	A > G, P
Furnishings	1.14 (0.14)	1.93 (0.07)	0.38 (0.14)	0.89 (0.26)	A > G, P
Substrate	1.05 (0.16)	2.00 (0.00)	0.23 (0.12)	0.67 (0.33)	A > G, P
Topography	1.03 (0.15)	1.87 (0.09)	0.23 (0.12)	0.78 (0.28)	A > G, P
Social grouping	0.86 (0.13)	1.33 (0.19)	0.31 (0.13)	0.89 (0.26)	A, P > G
Visual barrier	0.84 (0.15)	1.73 (0.15)	0.00 (0.00)	0.56 (0.24)	A > G, P
Exhibit size	0.81 (0.15)	1.60 (0.13)	0.00 (0.00)	0.67 (0.33)	A > P > G
Vegetation	0.73 (0.14)	1.53 (0.17)	0.00 (0.00)	0.44 (0.24)	A > G, P
Enrichment	0.43 (0.10)	0.87 (0.17)	0.08 (0.08)	0.22 (0.15)	A > G, P

*Note:* The maximum score per measure is 2. Mann–Whitney *U* tests show significant differences between assessment measure and zoo type. Commas between zoo types represent no significant difference.

a No significant difference found between zoo types.

### 3.2 Accredited Zoos

**Figure 1:**
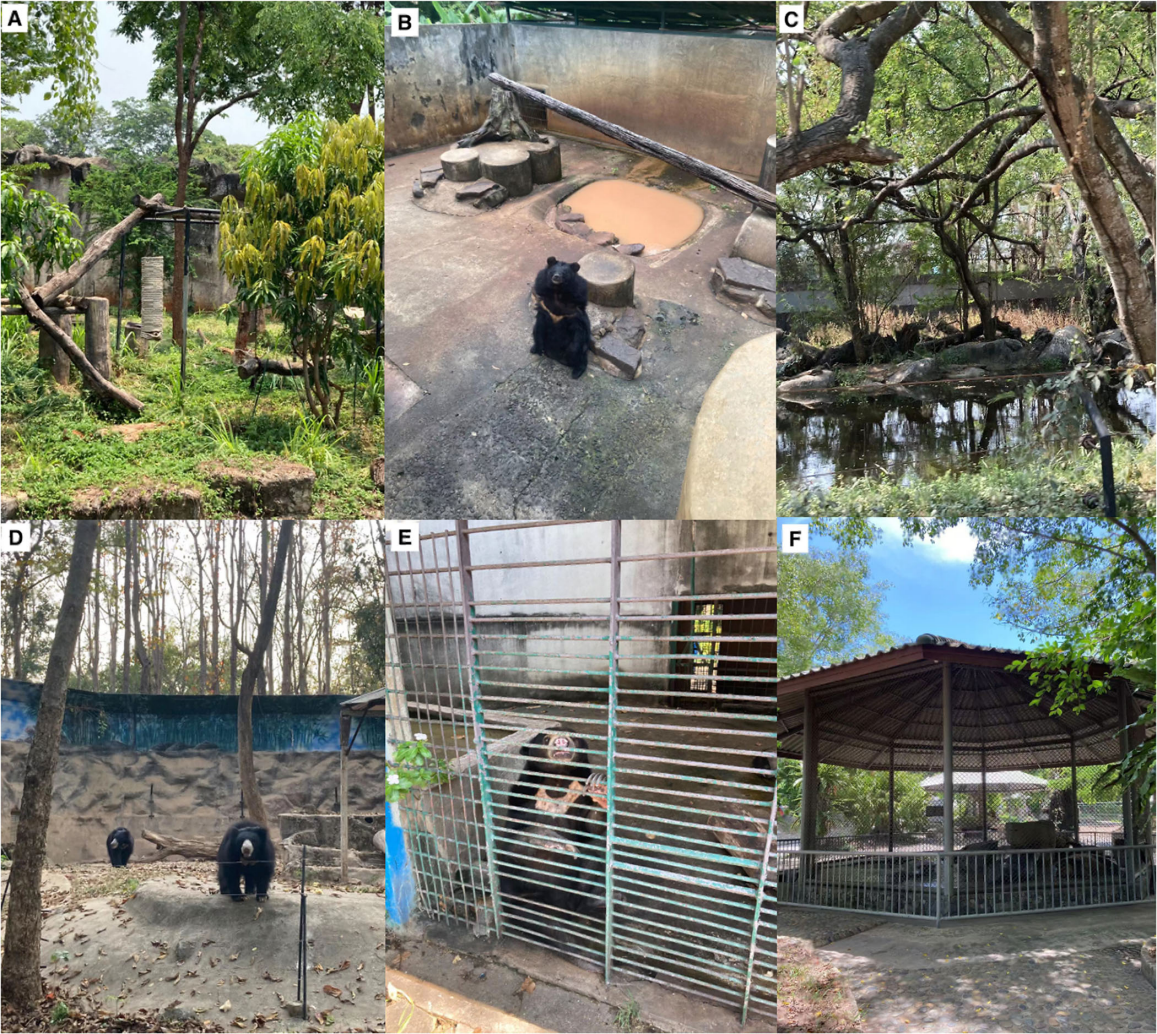
Photographs of bear exhibits with corresponding exhibit deficiencies (without data on water and back‐of‐house access) from Table 4: (A) occasional music from a nearby loudspeaker; (B) insufficient size, flat, concrete substrate, limited furniture, no vegetation, no visual barriers, pit‐style, no enrichment; (C) hotwire was only barrier containment, no enrichment and loud noise from the safari bus microphone; (D) minimum size, mainly flat, minimum furniture and shade, no vegetation, no visual barriers, occasional loud noise from a show; (E) insufficient size, flat, concrete substrate, no access to direct sunlight, completely shaded, poor ventilation, minimal furniture, no vegetation, no visual barriers, metal bars with an ineffective guardrail, no enrichment, substantial build‐up of faeces and food, bear housed alone; (F) insufficient size, flat, concrete substrate, no access to direct sunlight, completely shaded, loud noise from the nearby show, minimal furniture, no vegetation, no visual barriers (circular exhibit, bears viewable on all sides), no enrichment, dirty exhibit.

All 15 exhibits in accredited zoos met the AZA Bear Taxon Advisory Group (2019) minimum size between 279 and 465 m^2^, with 60% (*n* = 9) exceeding the recommended minimum size (Table 4 and Figure 1A,D). The exhibits' topography varied, with 87% (*n* = 13) having hills and rocky outcrops throughout the enclosure, which provided the bears' space equal or elevated to the visitor viewing level. The remaining two exhibits only had a minor variation in topography. In total, 87% (*n* = 13) of exhibits had a moat barrier; two exhibits (both in the same zoo) were pit‐style, as bears were displayed below the visitor viewing level. One of these exhibits did not safely contain the bear, who could have climbed up overgrown tree roots and out of the exhibit. Almost all exhibits (93%, *n* = 14) had multiple items of species‐appropriate furniture, such as caves, platforms, climbing structures, logs and ponds.

**Table 4:** Rank score of 37 exhibits (maximum score is 28), listing the main deficiencies within each exhibit.

Zoo	Number of bears	Main welfare problems	Total score
A1	3	Minimal enrichment. **Water in a pond, large exhibit and cannot see if there is separate drinking water. Has a back‐of‐house area but cannot see if it is accessible**	27
A1	2	Minimal enrichment. **Water in a pond, large exhibit and cannot see if there is separate drinking water. Has a back‐of‐house area but cannot see if it is accessible**	27
A2	2	Occasional music from a nearby loudspeaker. **Water in a pond, large exhibit and cannot see if there is separate drinking water. Has a back‐of‐house area but cannot see if it is accessible**	27
A2	2	Occasional music from loudspeaker. **Water in a pond, large exhibit and cannot see if there is separate drinking water. Has a back‐of‐house area but cannot see if it is accessible**	27
A3	1	Minimal enrichment. **Water in a pond, large exhibit and cannot see if there is separate drinking water. Has a back‐of‐house area but cannot see if it is accessible**	25
A4	3	Minimal enrichment, shelter available in one part of exhibit only. **Water in a pond, large exhibit and cannot see if there is separate drinking water. Has a back‐of‐house area but cannot see if it is accessible**	25
A4	1	Minimal enrichment. Bear housed alone. **Water in a pond, large exhibit and cannot see if there is separate drinking water. Back‐of‐house is inaccessible**	25
A3	3	Exhibit meets minimum size, minimal enrichment, and over‐crowding for exhibit size. **Water in a pond, large exhibit and cannot see if there is separate drinking water. Has a back‐of‐house area but cannot see if it is accessible**	25
A3	3	Exhibit meets minimum size, no enrichment, over‐crowding for exhibit size. **Water in a pond, large exhibit cannot see if additional water bowl. Has a back‐of‐house area but cannot see if it is accessible**	24
A5	2	Minimum vegetation, no enrichment, frequent loud noise from safari bus microphone. **Cannot see a water source. Back‐of‐house is not accessible**	23
A5	2	Minimum vegetation, no enrichment, loud noise from microphone**. Cannot see a water source. Back‐of‐house is not accessible**	23
P1	4	Hotwire was only barrier containment, no enrichment, and loud noise from the safari bus microphone. **Water in a pond, large exhibit and cannot see if there is separate drinking water. Has a back‐of‐house area but cannot see if it is accessible**	22
A6	2	Exhibit meets minimum size, mainly flat, minimal vegetation and enrichment, pit‐style. **Water in a pond and cannot see if there is separate drinking water. Back‐of‐house is not accessible**	21
A6	2	Exhibit meets minimum size, minimal vegetation and enrichment, pit‐style, the bear could climb out of exhibit due to the dry moat and proximity of tree. **Water in a pond, did not observe separate water source. Back‐of‐house is not accessible**	22
P2	3	Minimum shelter, vegetation, visual barriers and enrichment. Loud noise from safari bus microphones. **Water in a pond, large exhibit and cannot see if there is separate drinking water. Has a back‐of‐house area but cannot see if it is accessible**	20
A5	2	Minimum size, minimum vegetation, no enrichment, loud noise from safari bus microphone. **Water in a pond, large exhibit and cannot see if there is separate drinking water. Back‐of‐house is not accessible**	21
P1	9	No enrichment, hotwire as the only barrier, loud noise from safari bus microphones, over‐crowded for exhibit size. **Water in a pond, did not observe separate water source Back‐of‐house is inaccessible**	17
A5	2	Minimum size, mainly flat, minimum furniture and shade, no vegetation, no visual barriers, occasional loud noise from a show. **No water source observed. Back‐of‐house but is not accessible**	17
G1	1	Insufficient size, mainly flat, no vegetation, limited shade and furniture, no visual barriers, no enrichment, bear housed alone. **No water source observed. Back‐of‐house is not accessible**	14
G1	1	Insufficient size, flat, concrete substrate, no vegetation, limited shade and furniture, no visual barriers, no enrichment, bear housed alone. **Dirty moat, separate drinking source but dirty water. Back‐of‐house is not accessible**	13
G2	3	Insufficient size, mainly flat, no shade, no vegetation, limited furniture, no visual barriers, pit‐style, minimum enrichment, too many bears for exhibit size. **Dirty moat, separate drinking source but dirty water. Back‐of‐house is not accessible**	15
G1	1	Insufficient size, flat, no vegetation, limited shade and furniture, no visual barriers, no enrichment, bear housed alone. **Dirty moat, separate drinking source but dirty water. Back‐of‐house is not accessible**	13
P3	2	Insufficient size, flat, concrete substrate, no vegetation, no visual barriers, loud noises from animal shows. **Clean water in a pond and bowl. Back‐of‐house is not accessible**	11
G3	2	Insufficient size, flat, concrete substrate, no furniture, no vegetation, no visual barriers, pit‐style, no enrichment. **Dirty water in a bowl. Back‐of‐house is accessible**	9
G3	1	Insufficient size, flat, concrete substrate, no furniture, no vegetation, no visual barriers, no enrichment, bear housed alone. **Dirty water in a bowl. Back‐of‐house is accessible**	9
P4	1	Insufficient size, flat, concrete substrate, no furniture, no vegetation, no visual barriers, no enrichment, bear housed alone, **Dirty water in a bowl. Back‐of‐house is not accessible**	9
P3	1	Insufficient size, flat, concrete substrate, no vegetation, no visual barriers, frequent loud noise from nearby shows, no enrichment, bear housed alone. **Clean water in a bowl. Back‐of‐house is not accessible**	9
G3	1	Insufficient size, flat, concrete substrate, no shade, no furniture, no vegetation, no visual barriers, no enrichment, dirty exhibit, bear housed alone. **Dirty water in a bowl. Back‐of‐house is accessible**	9
G4	2	Insufficient size, flat, concrete substrate, poor ventilation, no furniture, no vegetation, no visual barriers, pit‐style, no enrichment, dirty exhibit. **Dirty water in a bowl. Back‐of‐house is not accessible**	9
G4	2	Insufficient size, flat, concrete substrate, poor ventilation, no furniture, no vegetation, no visual barriers, pit‐style, no enrichment, dirty exhibit. **Dirty water in a bowl. Back‐of‐house is not accessible**	9
P4	4	Insufficient size, flat, concrete substrate, poor ventilation, no furniture, no vegetation, no visual barriers, frequent loud noises from nearby animal shows, pit‐style, no enrichment, over‐crowded, **Dirty water from moat. Back‐of‐house is not accessible**	8
G4	1	Insufficient size, flat, concrete substrate, no shade, poor ventilation, no furniture, no vegetation, no visual barriers, pit‐style, no enrichment, dirty exhibit, bear housed alone. **Dirty water in a bowl. Back‐of‐house is accessible**	7
G5	1	Insufficient size, flat, concrete substrate, no access to direct sunlight, completely shaded, poor ventilation, no furniture, no vegetation, no visual barriers, no enrichment, dirty exhibit, bear housed alone. **Dirty water from small trough. Back‐of‐house accessible**	6
G5	1	Insufficient size, flat, concrete substrate, no access to direct sunlight, completely shaded, poor ventilation, no furniture, no vegetation, no visual barriers, no enrichment, dirty exhibit, bear housed alone. **Dirty water from small trough. Back‐of‐house accessible**	6
G6	1	Insufficient size, flat, concrete substrate, no access to direct sunlight, completely shaded, poor ventilation, minimal furniture, no vegetation, no visual barriers, metal bars with an ineffective guardrail, no enrichment, substantial build‐up of faeces and food, bear housed alone. **Dirty water in a bowl. No back‐of‐house**	6
P5	2	Insufficient size, flat, concrete substrate, no access to direct sunlight, completely shaded, loud noise from the nearby show, minimal furniture, no vegetation, no visual barriers (circular exhibit, bears viewable on all sides), no enrichment, dirty exhibit. **Dirty water in a bath, no separate drinking water. No back‐of‐house**	6
P5	1	Insufficient size, flat, concrete substrate, no access to sunlight, completely shaded, poor ventilation, loud noise from the nearby show, no furniture or vegetation, no enrichment, bear housed alone. **Empty water bowl (no pond). No back of‐house**	5

*Note:* The text highlighted in bold reports on the provision of water and back‐of‐house access and is not scored in the quantitative analysis. Zoo type is indicated by A (Accredited zoo), G (Government zoo) and P (Private zoo). The numbers following the letters A, G, and P represent different zoos within each category.

All exhibits except one contained vegetation, with 60% (*n* = 9) of exhibits having varied plants and shrubs throughout and the other 55% (*n* = 5) of exhibits having a moderate amount. One exhibit appeared to have vegetation, but access to the plants and shrubs was blocked by electric fencing. In total, 80% (*n* = 12) of exhibits provided suitable visual barriers in different areas of the exhibit, with only 7% (*n* = 1) of exhibits not providing any opportunity for the bears to hide. All exhibits (*n* = 15) had varied substrates, including grass, dirt and sand, and 100% (*n* = 15) of the exhibits were very clean. Only 13% (*n* = 2) of the exhibits had multiple enrichment items: hammocks, ropes, tyres, scratching posts and boomer balls. A further 60% (*n* = 9) of exhibits had only one or two manipulable items, whilst 27% (*n* = 4) of exhibits had no manipulable items at all. Just under one‐half of exhibits (47%, *n* = 7) had appropriate social grouping, whereby two or more bears were socially housed in large enough and resource‐sufficient exhibits. A further 40% (*n* = 6) of exhibits had two or more bears but were housed in exhibits without sufficient resources for all animals. Finally, 13% (*n* = 2) of exhibits housed bears alone.

The noise level was at an unacceptable level in 20% (*n* = 3) of exhibits due to sound from the tour guide's microphone on a safari bus. A further 27% (*n* = 3) of exhibits experienced moderate electronic noise, including one exhibit impacted by a nearby animal show and two exhibits located next to a loudspeaker that played repetitive music. All accredited zoos had a back‐of‐house, where access could not be confirmed in 53% (*n* = 8) of exhibits and 47% (*n* = 7) where we observed that access was closed. We observed ponds in 80% (*n* = 12) of the exhibits but not the remaining three exhibits (20%) in the same zoo. We could not see any drinking water receptacles in any exhibit, which may be due to the large size of the enclosures.

### 3.3 Government Zoos

Exhibits in 100% (*n* = 13) of government zoos were below the minimum size of 279 m^2^ (Table 4 and Figure 1B,E). Enclosures were completely flat in 77% (*n* = 10) of exhibits, with the remaining 23% (*n* = 3) having only a slight variation in topography. All exhibits safely contained the bears, but 23% (*n* = 3) had no guardrails. Over a third of exhibits (38%, *n* = 5) were pit‐style enclosures, whilst 54% (*n* = 7) of exhibits had metal bars. There were no guardrails in three of these exhibits. Exhibits were overall very barren, with 62% (*n* = 8) having no furniture and 38% (*n* = 5) of exhibits only providing very basic furnishing, such as a simple climbing structure or an elevated platform.

None of the 13 exhibits had any vegetation, nor did any of the exhibits provide visual barriers from visitors, conspecifics or animals in neighbouring exhibits. Aside from insufficient furniture and the absence of any vegetation, exhibits were generally so small that there was no place for the bear to hide. Most exhibits (77%, *n* = 10) had concrete floors, with 46% (*n* = 6) of exhibits scoring a zero for cleanliness as they had evidently not been cleaned for some time. Regarding the provision of enrichment, the only items that could be considered enrichment were three tyres in one exhibit (8%). Nine exhibits (69%) housed bears alone, and the remaining 31% (*n* = 4) of exhibits displayed more than one bear in a space without sufficient resources.

Noise was the highest scoring measure in government zoos, with 100% (*n* = 13) of exhibits scoring a two due to an absence of loudspeakers, microphones or sound systems that usually accompanied animal shows. Most (62%, *n* = 8) of the exhibits had a back‐of‐house area, with 56% (*n* = 5) having access. However, of the five exhibits with back‐of‐house access, these areas were generally very small. Some type of water source could be found in 92% (*n* = 12) of exhibits. This could be seen easily as the exhibits were small and uncomplex, but none of the water provided was clean. The water receptacles usually had algae growth, and water was brown or stagnant.

### 3.4 Private Zoos

Of the nine exhibits surveyed in private zoos, 33% (*n* = 3) exceeded the minimum size of 279 m^2^ (Table 4 and Figure 1C,F). The remaining 67% (*n* = 6) of exhibits were all below 50 m^2^. Just 22% (*n* = 2) of exhibits had varied topography with rocky structures and other furniture, whilst 44% (*n* = 4) were completely flat. An electric fence without backup systems served as the primary containment method in 33% (*n* = 3) of exhibits and scored a zero. Bears were safely contained in 67% (*n* = 6) of exhibits by guardrails, which prevented visitor contact. The metal bars used as the barrier are contrary to recommendations for the provision of good welfare and are inappropriate for public display. The provision of furniture scored poorly as only 22% (*n* = 2) had well‐furnished exhibits, and 33% (*n* = 3) had no furniture within the exhibit.

Most exhibits (67%, *n* = 6) had no vegetation, with only 11% (*n* = 1) in a safari setting having extensive vegetation throughout. More than half of the exhibits (55%, *n* = 5) did not provide any visual barriers for the bears, with only one exhibit providing opportunities for the bears to hide. Three exhibits (33%) had a variety of natural substrates, whilst the remaining 67% (*n* = 6) were concrete or tile. A majority of exhibits (55%, *n* = 5) were very clean, with only one 11% (*n* = 1) exhibit that was dirty due to large quantities of discarded food and faeces. Whilst 78% (*n* = 7) of exhibits had no enrichment items, 12% (*n* = 2) of exhibits had one boomer ball in one exhibit and three tyres in another. Three exhibits (33%) housed single bears, and a further 44% (*n* = 4) of exhibits housed more than two bears together, but the number of bears was too many for the resources within the exhibit. Only 22% (*n* = 2) of exhibits adequately housed two more or bears.

Noise was an issue for all exhibits because of the proximity to animal shows or the frequent noise from microphones from safari buses. There were no back‐of‐house areas in 22% (*n* = 2) of private zoos. We could not observe if a back‐of‐house was accessible in one exhibit (11%), with the remaining 67% (*n* = 6) of exhibits having no access to the back‐of‐house access area. In total, 78% (*n* = 7) of exhibits had ponds (including one moat). We observed that 22% (*n* = 2) of exhibits had no separate drinking water receptacle, and we could not see whether three more exhibits had a separate drinking water source. Of the 56% (*n* = 5) of exhibits where we could see a receptacle, two receptacles had dirty water, two were clean and one was empty.

## 4 Discussion

In this study, we developed a practical scoring framework to evaluate the environmental parameters of captive bears in Thailand as an indicator of welfare potential. We found that most exhibits failed to meet the physical and behavioural needs of bears due to largely small and barren spaces. This finding is alarming as it suggests that more than half of captive bears experience substandard welfare in zoos in Thailand. We also investigated whether the quality of bear exhibits correlated with the zoo type and found that accredited facilities provided significantly better exhibits. Therefore, the following discussion of the shortcomings recorded in exhibits predominantly refers to exhibits in government and private zoos. However, it is important to note that there is still room for improvement in accredited zoos, particularly the provision of enrichment.

### 4.1 Exhibit Assessments

Overall, the main issues that we identified in bear exhibits in Thai zoos have also been reported in bear exhibits in Polish Zoos (Maślak et al. 2016), in North America (Laidlaw et al. 2010) and Japanese bear parks (Wild Welfare 2015). The deficiencies outlined in those exhibits are generally the same: small exhibits, inappropriate substrate (usually concrete), no enrichment and absent or dirty water, illustrating that bears are still unsuitably housed in many countries worldwide. The inadequate exhibits found in our study are similar to those previously reported on other species in Thai zoos over the last decade. For example, Schmidt‐Burbach et al. (2015) found that 75% of macaques (*Macaca* spp.) and 99% of tigers (*Panthera tigris*) in private zoos were kept in poor conditions, usually in exhibits that were too small, with concrete substrate and poor furnishings. Similarly, most hornbills displayed in non‐accredited zoos were not housed in appropriate exhibits (Fourage et al. 2022). Further, multiple accounts of poor elephant welfare in many facilities throughout Thailand relate to how housing and husbandry are unacceptable to international standards (Kontogeorgopoulos 2009; Nijman 2014; World Animal Protection 2018). These examples further exemplify the need for legislative changes, including implementing zoo standards and strengthening animal welfare legislation coupled with better enforcement in the issuing and renewing of zoo licences.

A widespread problem identified from our assessments was the large number of exhibits that were insufficiently sized. Although a large exhibit size does not necessarily translate to a more complex exhibit (Browning and Maple 2019), having more space is crucial because larger spaces facilitate increased complexity (Veasey 2017). Smaller, unstimulating environments can cause stereotypies, especially in wide‐ranging animals such as bears (Clubb and Mason 2007). A study by Vickery and Mason (2004) of bears housed in the Department of National Park Wildlife Rescue and Breeding Centres (not open to the public) in Thailand found stereotypies in individuals housed in small and uncomplex environments with exhibit features similar to those we documented. Whilst behaviour was not recorded as part of our research, we did observe stereotypies in numerous bears in all zoo types.

The general barrenness of many exhibits can impact an animal's physical and mental welfare state in multiple ways. For example, bears are ‘hard‐wired’ to forage and should have their food placed in multiple areas within the exhibit (Carlstead et al. 1991; Poole 1992). Using concrete substrates prevents bears from digging or foraging, behaviours which wild bears perform for a significant portion of daily activity budgets (Ames 1999; Vickery and Mason 2004). The use of concrete also has implications on thermal comfort as this substrate retains heat for long periods (Morgan and Tromborg 2007). In hot countries such as Thailand, using concrete coupled with insufficient shade may result in poor thermoregulation. Aside from being uncomfortable, the hard surface can cause foot health issues, including pododermatitis, a condition relating to the inflammation of the paw (Collins 2014). Exacerbating the inability of bears to adequately thermoregulate is the fact that many exhibits did not include a sufficient bathing source and drinking water. This lack of basic care can contribute to heat stress (Rogers et al. 2021), exposure to toxins from water contaminated with cyanobacteria (Doster et al. 2014) and behavioural restrictions.

Another concern was the lack of vegetation and visual barriers, creating an inability for bears to hide or retreat from conspecifics or visitors. The fact that many of the bears on display were always visible, including some housed in pit‐style enclosures and without access to back‐of‐house areas, has long been recognised as an entirely inappropriate way to display captive bears (Carlstead and Seidensticker 1991; Ross 2006; Laidlaw et al. 2010; Podturkin 2022). Studies show that providing animals a choice in their degree of visibility and accessibility reduces stress. For example, providing unrestricted back‐of‐house access resulted in less stereotypic behaviour in polar bears (Ross 2006), reduced pacing and increased foraging in American black bears (Bruno et al. 2023) and decreased agitated behaviour in giant pandas (*A. melanoleuca*) (Owen et al. 2005). The issue of animal visibility presents a dilemma to zoo management in balancing the needs of animal welfare, the ability of keepers to monitor the animal safely and catering to visitors' desires to see animals within the exhibit (Hosey et al. 2013). However, zoos that do not prioritise animal welfare may prefer to make the animal as visible as possible to please visitors.

The provision of environmental enrichment (the lowest assessment scoring measure in this study) was poor across all types. Given the high cognitive abilities of bears, an insufficient or absent enrichment programme significantly decreases welfare due to the lack of opportunity to explore novel items and perform the natural behaviours encouraged in well‐planned enrichment activities (Carlstead et al. 1991; Altman 1999; Soriano et al. 2016). Whilst we acknowledge the possibility that we did not observe all incidences of enrichment because we were not present or enrichment was olfactory, even defining enrichment in basic terms as sensory and tactile manipulable items such as ropes, tyres and boomer balls, enrichment was still markedly scarce. However, some accredited zoos have collaborated with an animal welfare NGO to provide enrichment programmes for their bears, including scatter feeding and scent enrichment (Wild Welfare 2019). Nevertheless, based on the previous studies on other species (see Schmidt‐Burbach et al. 2015; Fourage et al. 2022) exhibit conditions and management practices of many of the non‐accredited zoos, it is also likely that no enrichment was provided.

The issue of social housing raised questions in our study. A majority of bears in government facilities were housed alone in small, barren exhibits. There are various reasons that could explain why bears were singularly housed, including management concerns about introducing new bears to each other (many bears are either rescued or confiscated) or the fact that housing bears together to provide social opportunities may not even be a consideration. It is unlikely that bears were housed alone primarily for their welfare needs due to other negative housing aspects. Bears can be socially housed if exhibits have sufficient resources and retreat opportunities for all animals to prevent conflict (Maślak et al. 2016). Integration should consider factors such as medical issues, time of year, behaviour appetite and size of enclosure (Animals Asia 2017). However, since many of the exhibits assessed in our study do not provide sufficient size or resources, the question is whether bears could still benefit from social contact despite inadequate resources. Tan et al. (2013) found that Malayan Sun Bears housed indoors in very small and barren enclosures interacted more with conspecifics within their exhibit than bears housed outdoors. They suggest that this was because the barrenness of the exhibit environment meant that all the stimulation bears had was each other's company. Arguably, some bears in relatively barren exhibits could still benefit from being socially housed, albeit with obvious increased risk.

### 4.2 Differences Between Exhibits and Different Zoo Types

A key finding of our study was the notable difference in exhibit quality between zoo types, raising questions on the impact of institutional priorities and objectives on welfare provisioning. Overall, accredited zoos had good quality exhibits that provided the environmental resources necessary for bears to attain a higher welfare potential. The higher scores achieved by the accredited zoos in this study likely reflect the management's willingness to provide good animal welfare. Such management willingness can be illustrated by the improvement of several zoos in this study that improved exhibit conditions following recommendations as part of an assessment process for membership in the Southeast Association of Zoos and Aquariums (Agoramoorthy 2004). The degree to which welfare is impacted by the zoo association's welfare requirements and the incentive to have accreditation status, along with the importance of accreditation status to the Thai public, is unknown and deserves further research. In western societies, accreditation status helps bolster a zoo's social license to operate, reassuring the public that standards are being met (Veasey 2022). Nevertheless, it is important to highlight that accreditation does not always translate to being a good zoo in all aspects (Barber et al. 2010). For instance, one of the accredited zoos in this study still provides a lion (*Panthera leo*) and tiger show where the animals must perform unnatural behaviours such as walking bipedally.

Here, it is worth considering the reasons behind Thailand's lack of accredited private zoos. As discussed earlier, many Thai zoos have been widely criticised for the exploitative nature of their activities and welfare (Schmidt‐Burbach et al. 2015; Paddock 2019; Ratcliffe 2020; World Animal Protection 2020; Cowan 2022). Because some species, such as bears, can live relatively long lives even in substandard conditions, this can incorrectly imply that these animals do not have complex needs (Law and Reid 2010; Sergiel and Maślak 2014). Additionally, some private zoos may believe that applying for accreditation is detrimental to their interests (usually financial) if they must improve welfare to meet accreditation standards, similar to observations made by Keulartz (2015) and Veasey (2022). Welfare and commercial interests do not need to be in conflict; in western zoos, the provision of better welfare is considered better for profitability because poor welfare standards can cause public outcry and are thus a liability for an institution (Veasey 2022). In countries in the Global South such as Thailand, animal welfare is recognised differently in policy and societal expectations than in developed countries due to the differences in knowledge and attitudes (Sinclair et al. 2022; Parlasca et al. 2023). However, knowledge, attitudes and perceptions can be changed. For instance, in Thailand, behaviour change campaigns have resulted in ivory reduction (Wild Aid 2022). In addition to the implementation of any regulatory measures, it is necessary to alter the public's behaviour so that people do not visit dysfunctional facilities.

Zoo licensing requirements do not stipulate standards of care, which also likely contributes to the numerous substandard zoos within the country (Schmidt‐Burbach et al. 2015; Beastall et al. 2016; World Animal Protection 2018). Furthermore, existing animal welfare legislation needs to be strengthened as the extent of protection afforded to captive wildlife is ambiguous (Agoramoorthy and Hsu 2005; Beastall et al. 2016). Consequently, providing good animal welfare is not obligatory in Thai zoos, which enables the continuation of poor welfare.

Government zoos face other types of challenges that impact their ability to provide a good level of care for bears. Perhaps the most pressing issue is the large government budget cuts. The budget of the Department of National Parks was reduced by over 60% in 2021/2022, at a time when they were reportedly responsible for 222 Asiatic black and sun bears (Anon 2022). Department of National Park Wildlife Rescue and Breeding Centres are obligated to receive confiscated or rescued wildlife, but without sufficient resources, there is pressure to suitably provide for animals' needs (Cadigan 2013; Nijman 2014; Paddock 2019; Wonruang 2020). A further factor is a reluctance to use euthanasia as a management tool in Thai zoos to manage the number of animals (Agoramoorthy and Harrison 2002; Fuller 2013). Euthanasia is not widely practised in Thai society due to prevalent Buddhist beliefs (Chaichoreon and Ratanakul 1998). As a result, facilities struggle to finance the basics, such as animal feed and staff salaries. Due to the unpredictability of the intake of confiscated and rescued animals, facilities cannot effectively plan for the animals they must accommodate. Compounding the pressure is that the Department of National Parks is required to hold confiscated animals for up to 5 years during legal proceedings, significantly contributing to the overcrowding of facilities (Wonruang 2020).

However, Barber et al. (2010) contend that despite the difficulties faced by individual zoos, animal welfare should still not be compromised. There are ways to improve the quality of exhibits (and a bear's quality of life) without being too cost‐prohibitive. For example, drinking receptacles should always be clean, with water available. Enrichment items are often freely available onsite and browse, coconuts, and leaves can be placed on concrete floors. Manipulable items, including ropes, hammocks, tyres, log platforms and logs for scratching, should be offered. Nevertheless, as highlighted by Ward et al. (2020) in their paper on challenges faced by zoos in developing countries, if staff are not equipped with knowledge that these simple items are necessary to improve bear welfare in the first place, then the above suggestions are meaningless.

We recognise that only assessing environmental parameters represents a glimpse of an animal's welfare potential and that other factors, such as an animal's life history, personality, and physical health, need to be considered. We also recognise that some parameters, especially enrichment and the presence or absence of noise, may vary depending on the time the assessor visited. As such, our assessment represents a snapshot view. Yet, despite the limitations of this study in only assessing front‐of‐house measures and without detailed information on individual animals or zoo management practices, our results can still be used to provide baseline data on the current state of captive bear welfare in Thailand.

## 5 Conclusion

We aimed to identify the welfare potential for bears housed in different zoo types in Thailand by assessing the environmental parameters within bear exhibits. Our findings showed that many bears were displayed in exhibits, which reduced the potential for them to experience good welfare. The main issues we uncovered were exhibits that were too small and without adequate furniture, vegetation and enrichment. Exhibits in accredited zoos were significantly better than in government and private zoos, highlighting the need for zoo associations and accredited zoos to actively work with non‐accredited zoos to increase standards within the zoological community. Furthermore, our simple assessment method can be helpful for zoos in countries with limited assessment resources. This study highlights the urgent need to improve the monitoring of zoo collections and the need to improve animal welfare legislation and implement robust zoo standards.

## Supporting information

Fourage (2024) ‐ S1.docx.

## Data Availability

The data that support the findings of this study are available from the corresponding author upon reasonable request.
