# Supplementary material for: Environmental Parameters as Indicators of Bear Welfare
Source: Zoo Biol. 2025 Jul 14;44(5):480–94. doi: 10.1002/zoo.70010 (PMC12513127; doi:10.1002/zoo.70010)
Supplement: Supplementary file 1 — Fourage (2024) ‐ S1.docx. [file ZOO-44-480-s001.docx]

| **Measure** | **Key literature** |
| --- | --- |
| **Size** | - Minimum exhibit size recommendations often differ among national and regional (EU) legislation, zoo associations and other husbandry guidelines (Hosey et al., 2013). - Bears have large home ranges; a positive correlation exists between the size of a bear's home range and increased pacing in captivity (Clubb & Mason, 2003). - AZA husbandry guidelines for Malayan sun bears *(Helarctos malayanus),* and sloth bears *(Melursis ursinus)* stipulate a minimum size of 279-465m^2^. Ideally, new enclosures should be larger than 465 m^2^ per pair of bears housed (AZA, 2019). - 500m^2^ is recommended for a pair of Asiatic black bears *(Ursus thibetanus)* or Malayan sun bears (Animals Asia, 2017). - Exhibit size is important, but so is exhibit complexity (Browning & Maple, 2019). More extensive exhibits allow animals more control over whether they want to be close to conspecifics and provide the space for more complex environments (Ross et al., 2009). |
| **Permanent furniture** | - Bear exhibits should include climbing structures, raised platforms, caves/dens, and various resting and sleeping areas sufficient for all animals within the exhibit to prevent conflict and aggression (GFAS, 2013; Animals Asia, 2017; AZA, 2019). - Exhibits should have a permanent water source for bathing/swimming (AZA, 2019). - All bear species need a pool for bathing/swimming (GFAS, 2013; Animals Asia, 2017; AZA, 2019). |
| **Shelter and shade** | - All bears within an exhibit should have shelter from inclement weather (GFAS, 2013; AZA, 2019). - Shade should be available for all bears in different parts of the exhibit to allow for differences in microclimatic variations (Lorenzo, 2009). - Shelter and shade can be provided by roof overhangs, shade sails, dens, and caves (GFAS, 2013). - Heat stress and thermal comfort (Wark et al., 2020) - Ocular pathologies and squamous cell carcinomas have been observed in Malayan sun bears that have been over-exposed to the sun; thus, abundant shade is crucial for this species (Mylniczenko et al., 2005). |
| **Substrate** | - Variable substrates are essential to encourage natural behaviours such as foraging and digging (GFAS, 2013; AZA, 2019). - Natural substrates help with thermoregulation, whilst concrete can retain heat (Morgan & Tromborg, 2007). - Concrete substrates can cause physical discomfort due to the hard surface and can cause injuries by compromising foot and joint health (Collins, 2015). |
| **Vegetation** | - Various plants and vegetation should be planted throughout the exhibit to increase complexity (GFAS, 2013; AZA, 2019). - Trees, shrubs, bushes, and grasses are highly recommended exhibit elements that greatly enhance natural feeding and foraging opportunities (AZA, 2019). - Care should be taken to ensure that trees and vegetation are non-toxic (Lorenzo, 2009). - Plants and vegetation are helpful as both visual and noise barriers (de Azevado et al., 2023). |
| **Exhibit barrier** | - As bears are excellent escape artists, it is imperative that the exhibit is secure and escape-proof. Objects such as trees, rocks and other structures should not be placed near the exhibit barrier to prevent escape (Kolter et al., 1997). - Hotwire should not be used as the primary containment method in case of failure (Animals Asia, 2017). - A guardrail is necessary to protect people from getting bitten or mauled and to help prevent visitors from feeding the bears (Laidlaw et al., 2010). - Bears should not be viewed on all four sides – one or two sides maximum (Kolter et al., 1997). |
| **Environmental Enrichment** | - A comprehensive enrichment programme is essential for bears to perform more natural behaviours in captivity (Law and Reid, 2010). - Even in highly naturalistic enclosures, animals can still perform stereotypic behaviour due to individual histories and personalities (Swasigood & Shepherdson, 2005). - Different enrichment strategies should be implemented and changed daily (feeding, sensorial, social) (Animals Asia, 2017). - Bears need a variety of enrichment items that are regularly changed. Examples include boomer balls, browse (bamboo, leaves), bedding materials (hay and straw), firehose toys (hammocks), feeder toys (balls, tubes), fabrics, ice treats, paper (boxes, feed sacks, cardboard) (Animals Asia, 2017). |
| **Topography** | - The topography within the exhibit should be varied to provide complexity and opportunities for the bear to see out and beyond the exhibit (Law and Reid, 2010). - Bears should never be exhibited below the visitor level as this can stress the animal (Laidlaw et al., 2010; Law & Reid, 2010). - The use of furniture to create vantage points for bears to see out of their exhibit is important for welfare (Lorenzo, 2009). |
| **Visual barrier** | - Visual barriers give animals a choice to retreat from both conspecifics and visitors (de Azevado et al., 2023). - Results from visitor effect studies are mixed; some suggest that higher visitor numbers do not negatively impact behaviour in some bear species (Bernstein-Kurtcyz et al., 2021; Podturkin, 2022). Other studies report sensitivity to visitor presence in giant pandas and sun bears (Owen et al., 2004; Owen et al., 2014); increased stereotypies in brown bears (Soriano et al., 2013) - Unrestricted access to off-exhibit areas reduces stereotypies in polar bears *(Ursus maritimus)* (Ross, 2006); brown bears *(Ursus arctos)* (Podturkin, 2022); reduced agitated behaviour in giant pandas (*Ailuropoda melanoleuca)* (Owen et al., 2005). |
| **Cleanliness** | - Faeces, old food, and browse should be removed daily. Grass and soil substrates should be spot cleaned (Animals Asia, 2017). - Excessive cleaning with disinfectant should be avoided as over-sanitisation removes interesting smells and can cause irritation (Hosey et al., 2013. - Food and water bowls should be cleaned daily (AZA, 2019). |
| **Water** | - A separate drinking water receptacle should be provided as bathing water can get filthy if not properly filtered or if there is inadequate freshwater flow (GFAS, 2013; AZA, 2019). |
| **Social grouping** | - Bears are considered predominantly solitary in the wild (Stirling, 1993), yet this solitary living may be due to resource competition. - Tigers (*Panthera tigris*) have also been considered solitary in the wild but more evidence is suggesting that they have greater social plasticity that previously imagined (Holland et al., 2023). - Housing bears together can reduce stress and stereotypies by providing opportunities for social interactions and companionship (Animals Asia, 2017). - It is important to consider sex, age, personality, and hierarchies when determining appropriate social groups (Animals Asia, 2017). |
| **Noise** | - Noise from zoo visitors negatively impacts the welfare of several zoo-housed species (Quadros et al., 2014). - Loud environmental noise (such as events and construction) can cause stereotypic and/or agonistic behaviour in bears when noise levels exceed tolerance (AZA, 2019). - Studies on bear hearing capabilities are limited to polar bears' sensitive hearing in the polar bear over a wide frequency range. Therefore, anthropogenic noise for captive bears must be carefully considered (Nachitgall et al., 2007). - Giant pandas showed individual-specific behavioural and physiological changes when exposed to demolition noise in a zoo (Powell et al., 2006); giant pandas increased agitated behaviour with louder ambient noise (Owen et al., 2004). |
| **Ventilation** | - Bears have a highly evolved sense of smell (Sergiel & Van Horn, 2019). Poorly ventilated spaces can cause olfactory discomfort, especially if the exhibit is not clean, but it can also prevent interesting, new smells from entering the exhibit (Hosey et al., 2013). - Poor ventilation can also cause thermoregulation discomfort by preventing the flow of fresh air (Morgan & Tromborg, 2007). - Cross-ventilation in exhibits is important, especially in warmer climates (GFAS, 2013). |
| **Light** | - Bears should have access to natural light, considering the intensity, duration, and spectrum of light, which can impact behaviour (AZA, 2019). - Insufficient access to UVB light impacts the ability of animals to synthesize vitamin D (Hosey et al., 2013) and causes associated health problems, including rickets (Holick, 2002). |

**References**

AZA Bear Taxon Advisory Group. (2019). https://assets.speakcdn.com/assets/2332/sun_and_sloth_bear_care_manual_2019.pdf

Animals Asia. (2017)*.* <https://www.animalsasia.org/assets/pdf/AA-Handbook-EN.pdf>

Bernstein-Kurtycz, L. M., Koester, D. C., Snyder, R. J., Vonk, J., Willis, M. A., & Lukas, K. E. (2021). 'Bearly' changing with the seasons: Bears of five species show few behavioural changes across seasons and at varying visitor densities. *Animal Behavior and Cognition, 8*(*4*), 538-557. https://doi.org/10.26451/abc.08.04.07.2021

Browning, H. & Maple, T.L. (2019). Developing a Metric of Usable Space for Zoo Exhibits. *Frontiers in Psychology,* 11:10:791. https://doi.org/10.3389/fpsyg.2019.0079

Clubb, R., & Mason G. (2003). Captivity effects on wide-ranging carnivores. *Nature,* 425, 473.

Collins, D. M. (2014). Urisdae. In: (eds) Miller, R.E. & Fowler, M.E. *Zoo and Wild Animal Medicine.* Volume 8. Elsevier Saunders, 498-508.

de Azevedo, C.S.; Cipreste, C.F.; Pizzutto, C.S.; & Young, R.J. (2023). Review of the Effects of Enclosure Complexity and Design on the Behaviour and Physiology of Zoo Animals. *Animals,* *13*, 1277. https://doi.org/10.3390/ ani13081277

Global Federation of Animal Sanctuaries (GFAS). (2013). https://sanctuaryfederation.org/wp-content/uploads/2017/09/Bear-StandardsJune2013HA.pdf

Holick, M.F. (2002). Vitamin D: Importance for Bone Health, Cellular Health and Cancer Prevention. In: Holick, M.F. (ed.), Biologic Effects of Light 2001. Springer. <https://doi.org/10.1007/978-1-4615-0937-0_16>

Holland, A.; Galardi, E.G.; Fabbroni, M.; Hashmi, A.; Catinaud, J.; Preziosi, R.; Brereton, J.E.; Quintavalle Pastorino, G. Exploration of Social Proximity and Behavior in Captive Malayan Tigers and Their Cubs. *Animals* **2023**, *13*, 1040. https://doi.org/10.3390/ ani13061040

Hosey, G. R., Melfi, V., & Pankhurst, S. (2013*). Zoo animals: Behaviour, Management and Welfare* (2nd ed.). Oxford University Press.

Morgan, K & Tromborg, C. (2007). Sources of stress in captivity. *Applied Animal Behaviour Science,* 102. <https://doi.org/10.1016/j.applanim.2006.05.032>

Kolter, L., Kamphorst, N. F., & Ruven S. A. W. (1997). The design of new bear facilities. In J.G. Pickard (Ed.), *The Australasian husbandry manual for the Malayan sun bea*r (*Helarctos malayanus malayanus*) (pp. 7–32). Sydney, Australia: Wellington Zoo and the Australasian Regional Association of Zoological Parks and Aquaria (ARAZPA).

Nachtigall, P.E., Supin, A.Y., Amundin, M., Röken, B., Møller, T., Mooney, T.A., Taylor, K.A., & Yuen, M. (2007). Polar bear Ursus maritimus hearing measured with auditory evoked potentials. *Journal of Experimental Biology,* 210(7):1116-22. https://doi.org10.1242/jeb.02734

Law, G., & Reid, A. (2010). Enriching the lives of bears in zoos. *International Zoo Yearbook,* 44: 65–74.

Lorenzo, S. (2009). https://aszk.org.au/wp-content/uploads/2020/05/Mammals.-Brown-Bear-2009SL.pdf

Mylniczenko, N. D., Manharth, N. D., Clayton, L. A., Feinmehl, R., & Robbins, M. (2005). Successful treatment of mandibular squamous cell carcinoma in a Malayan sun bear (*Helarctos malayanus*). *Journal of Zoo and Wildlife Medicine, 36*(2), 346–348.

Owen, M.A., Swaisgood, R.R., Czekala, N.M., Steinman, K., & Lindburg, D.G. (2004). Monitoring stress in captive giant pandas (Ailuropoda melanoleuca): Behavioral and hormonal responses to ambient noise. *Zoo Biology,* 23. 147 - 164. https://doi.org/10.1002/zoo.10124

Owen, M.A, Swaisgood, R.R., Czekala, N.M. & Lindburg, D.G. (2005). Enclosure choice and well-being in giant pandas: Is it all about control? *Zoo Biology, 24*, 475–481. https://doi.org/10.1002/zoo.20064

Podturkin, A.A. (2022). Behavioral Changes of Brown Bears (*Ursus arctos*) during COVID-19 Zoo Closures and Further Reopening to the Public. *Journal of Zoological and Botanical Gardens, 3*: 256–270. https://doi.org/10.3390/ jzbg3020021

Powell, D., Carlstead, K., Tarou, L., Brown, J., & Monfort, S. (2006). Effects of construction noise on behavior and cortisol levels in a pair of captive giant pandas (Ailuropoda melanoleuca). *Zoo Biology,* 25. 391 - 408. https://doi.org/10.1002/zoo.20098

Quadros, S., Goulart, V., Passos, L., Vecci, M., & Young, R. (2014). Zoo visitor effect on mammal behaviour: Does noise matter? *Applied Animal Behaviour Science*, 156. http://doi.org/10.1016/j.applanim.2014.04.002

Ross, S.R. (2006). Issues of choice and control in the behaviour of a pair of captive polar bears (Ursus maritimus). *Behavioural Processes,* 73(1):117-20. https://doi.org10.1016/j.beproc.2006.04.003

Ross, S.R., Schapiro, S.J., Hau, J., & Lukas, K.E. (2009). Space use as an indicator of enclosure appropriateness: A novel measure of captive animal welfare. *Applied Animal Behaviour Science, 121*, 42–50.

Stirling, I., Kirshner, D & Knight, F. (1993). Bears Rodale Press.

Sergiel, A., Van Horn, R.C. (2019). Bear Sensory Systems. In: Vonk, J., Shackelford, T. (eds) Encyclopedia of Animal Cognition and Behavior. Springer, Cham. <https://doi.org/10.1007/978-3-319-47829-6_1745-1>

Soriano, A., Vinyoles, D., & Maté, C. (2013). The Influence of Visitors on Behaviour and the Use of Space in two Species of Ursids: a Management Question? *International Zoo News,* 60(5): 341-356.

Swaisgood, R.R., & Shepherdson, D.J. (2005). Scientific approaches to enrichment and stereotypies in zoo ani- mals: What's been done and where should we go next? *Zoo Biology,* 24: 499–518.

Wark, J., Wierzal, N., & Cronin., K. (2020). Mapping Shade Availability and Use in Zoo Environments: A Tool for Evaluating Thermal Comfort. *Animals,* 10:1189. https: doi//org.10.3390/ani10071189
